# Supplementary material for: Transcriptome Dynamics Underlying Planticine®-Induced Defense Responses of Tomato (Solanum lycopersicum L.) to Biotic Stresses
Source: Int J Mol Sci. 2023 Mar 30;24(7):6494. doi: 10.3390/ijms24076494 (PMC10095179; doi:10.3390/ijms24076494)

Supplementary Figure S3. Visualization of KEGG MAPK signaling pathway - plant pathway. Diffentially expressed genes (DEGs) were indicated in green boxes.

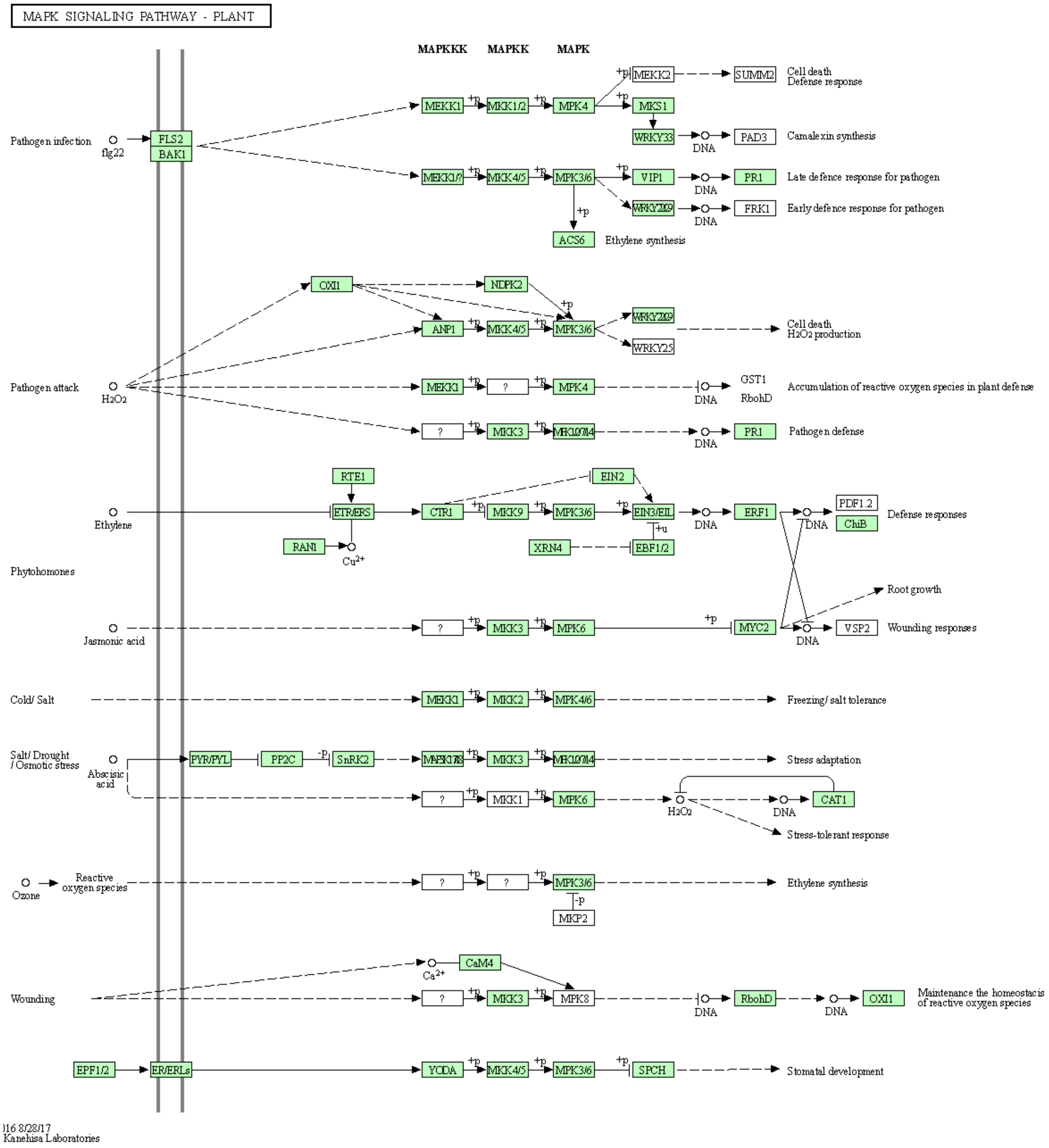

Supplement: Supplementary file 1 [file ijms-24-06494-s001.zip › Supplementary Figure S3_MAPK signaling pathway.pdf]
