# Supplementary material for: Transcriptome Dynamics Underlying Planticine®-Induced Defense Responses of Tomato (Solanum lycopersicum L.) to Biotic Stresses
Source: Int J Mol Sci. 2023 Mar 30;24(7):6494. doi: 10.3390/ijms24076494 (PMC10095179; doi:10.3390/ijms24076494)
Supplement: Supplementary file 1 [file ijms-24-06494-s001.zip › Supplementary Table S1_Sequencing statistics.pdf]

Supplementary Table S1. Sequencing statistics of 6 transcriptome libraries and mapping details

Treatment	Biological replicate	Reads	Bases (Gbp)	GC (%)	Q30 (%)	Clean reads	Total mapped to tomato genome	Uniquely mapped to genes	Multiple mapped to genes
Control	1	51,362,287	7.2	43	96,9	51,359,636	49,413,106 (96.21%)	43,088,787	1,042,384
	2	43,216,573	6.1	43	96.6	43,214,100	41,839,892 (96.82%)	36,666,846	857,894
	3	46,403,101	6.6	43	96.6	46,400,573	44,706,952 (96.35%)	38,941,574	938,338
Planticine®	1	46,963,609	6.6	42	96.7	46,960,150	45,283,673 (96.43%)	39,577,103	924,448
	2	47,960,977	6.8	42	96.7	47,957,642	46,221,575 (96.38%)	40,492,001	917,296
	3	53,281,554	7.5	42	96.6	53,277,130	51,508,329 (96.68%)	45,410,800	1,016,350
	Average	48,198,017	6.8			48,194,872			
	Total	289,188,101	40.8			289,169,231			

GC – the overall %GC of all bases in all sequences, Q30 – ratio of bases that have Phred quality score over 30, Clean reads refer adaptors trimmed and low-quality sequence filtered reads
