# Supplementary figures and images for: Transcriptome Dynamics Underlying Planticine®-Induced Defense Responses of Tomato (Solanum lycopersicum L.) to Biotic Stresses
Source: Int J Mol Sci. 2023 Mar 30;24(7):6494. doi: 10.3390/ijms24076494 (PMC10095179; doi:10.3390/ijms24076494)

**Supplementary Figure S1.** Pearson correlation analysis between all biological RNA samples

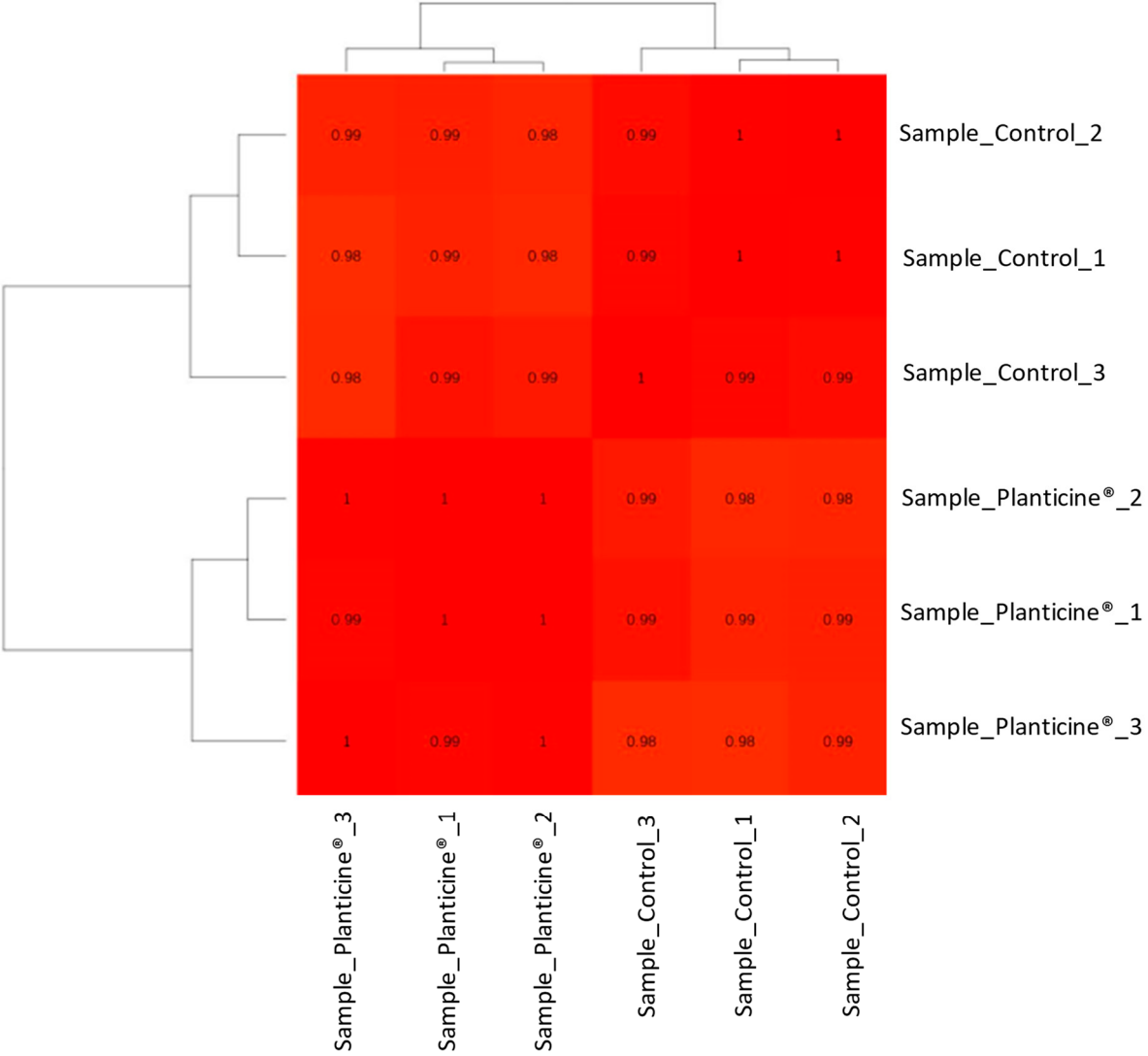

Supplement: Supplementary file 1 [file ijms-24-06494-s001.zip › Supplementary Figure S1_Pearson correlation.pdf]

34626 11/18/19  
(c) Kanehisa Laboratories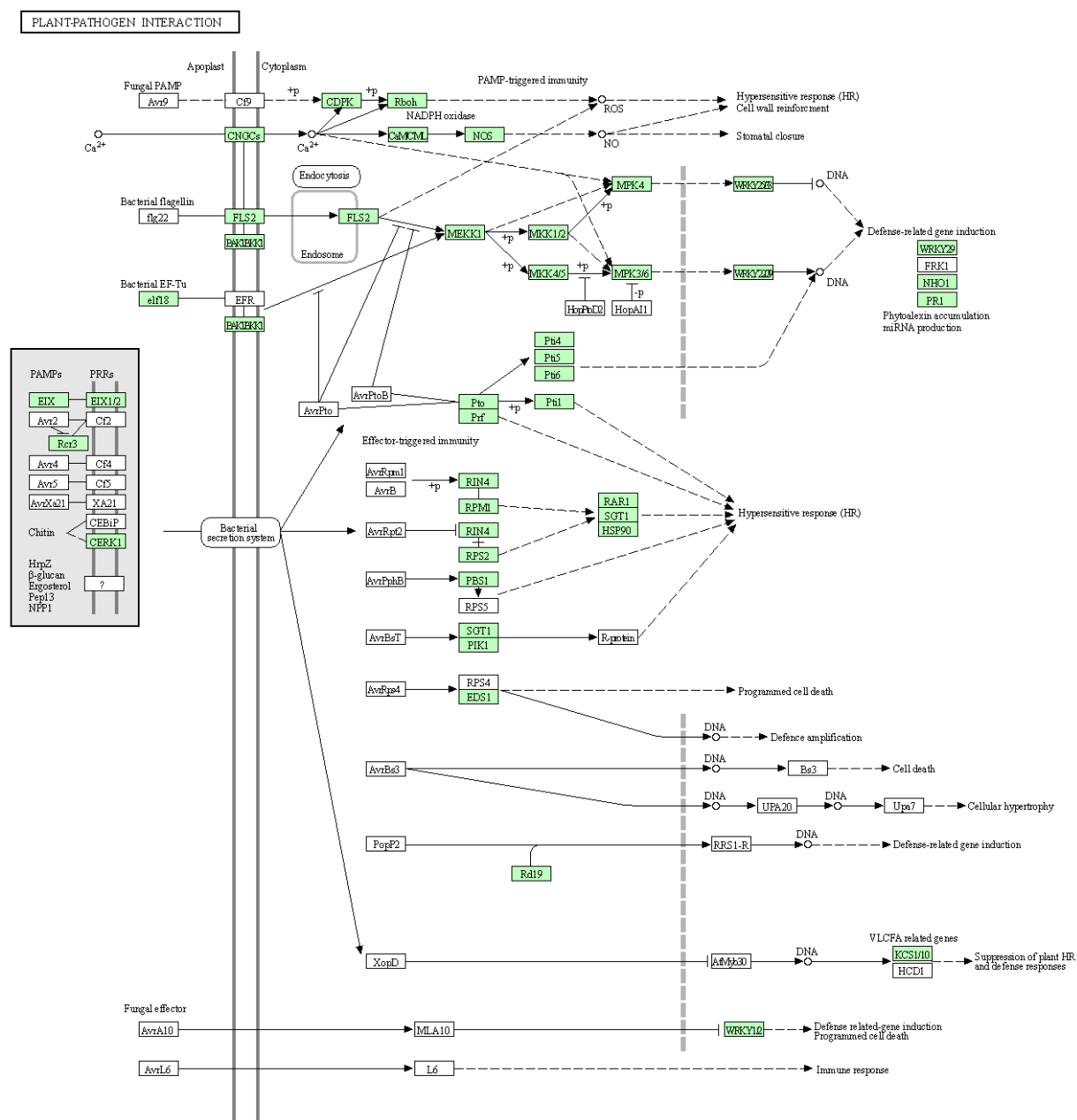

Supplement: Supplementary file 1 [file ijms-24-06494-s001.zip › Supplementary Figure S2_Plant-pathogen interaction pathway.pdf]
